# Health and Welfare in Dutch Organic Laying Hens

**DOI:** 10.3390/ani4020374

**Published:** 2014-06-20

**Authors:** Monique Bestman, Jan-Paul Wagenaar

**Affiliations:** Louis Bolk Institute, Hoofdstraat 24, 3972 LA, Driebergen, The Netherlands; E-Mail: j.wagenaar@louisbolk.nl

**Keywords:** organic, free range, poultry health, poultry welfare, feather pecking, vent pecking, mortality, keel bone deformations, foot sole lesions, comb color

## Abstract

**Simple Summary:**

Data on animal health and welfare and farm management during rearing and laying periods were collected from 49 flocks of organic laying hens in the Netherlands to establish how farms performed in terms of animal health and welfare and which factors affected health and welfare.

**Abstract:**

From 2007–2008, data on animal health and welfare and farm management during rearing and laying periods were collected from 49 flocks of organic laying hens in the Netherlands. Our aim was to investigate how organic egg farms performed in terms of animal health and welfare and which farm factors affected this performance. The flocks in our study were kept on farms with 34 to 25,000 hens (average 9,300 hens). Seventy-one percent of the flocks consisted of ‘silver hybrids’: white hens that lay brown eggs. Fifty-five percent of the flocks were kept in floor-based housing and 45% of the flocks in aviaries. No relation was found between the amount of time spent outdoors during the laying period and mortality at 60 weeks. Flocks that used their outdoor run more intensively had better feather scores. In 40% of the flocks there was mortality caused by predators. The average feed intake was 129 g/day at 30 weeks and 133 g/day at 60 weeks of age. The average percentage of mislaid eggs decreased from three at 30 weeks to two at 60 weeks. The average mortality was 7.8% at 60 weeks. Twenty-five percent of the flocks were not treated for worms in their first 50 weeks. Flubenol^©^ was applied to the flocks that were treated. Ten percent of the flocks followed Flubenol^©^ instructions for use and were wormed five or more times. The other 65% percent were treated irregularly between one and four times. Sixty-eight percent of the flocks showed little or no feather damage, 24% showed moderate damage and 8% showed severe damage. The feather score was better if the hens used the free-range area more intensely, the laying percentage at 60 weeks was higher, and if they were allowed to go outside sooner after arrival on the laying farm. In 69% of the flocks, hens had peck wounds in the vent area: on average this was 18% of the hens. Keel bone deformations were found in all flocks, on average in 21% of the birds. In 78% of the flocks, an average of 13% of the hens had foot-sole wounds, mostly a small crust. Combs were darker in flocks that used the range area more intensively. More fearful flocks had lighter combs. We conclude that organic farms are potentially more animal friendly than other poultry systems based on the animal welfare benefits of the free range areas. However, we also observed mortality rates, internal parasites, keel bone deformities, and foot sole lesions on organic farms that were comparable to or worse than in other husbandry systems. It is unclear whether these ‘remaining’ problems can be attributed to housing or if they are the result of keeping high productive genotypes in an artificial environment. Organic farms use the same high productive genotypes as other husbandry systems.

## 1. Introduction

The organic egg sector in the Netherlands increased from 150,000 hens on 40 farms in 2001 [1] to 2.1 million hens on 194 farms in 2011 [2]. The most important features of Dutch organic egg production are [3] a maximum group size of 3,000 birds, six birds per m^2^ indoors, 4 m^2^ per bird outdoors, 18 cm perch per bird, and one third of indoor floor surface covered with litter. Moreover, the hens are given organically grown feed. In 2008, this was 90% organic. In the Netherlands, there is an additional organic hen rearing regulation. Its most important features are a maximum stocking density of 24 birds per m^2^ during the first seven weeks and ten birds per m^2^ between 7–18 weeks of age, 7 cm perch per bird from seven weeks onwards, and access to free range starting from eight weeks. The main thing organic egg production has in common with free-range production is the free-range area. The main differences are the number of animals per m^2^ (six in organic and nine in free-range), organic ingredients in the feed, and farm size (up to 18,000 in organic and up to 40,000 in free range).

Many characteristics of organic poultry husbandry apply to loose housing in general. In the near future, we expect that the number of egg production systems with loose housing will further increase. In Europe, traditional cages are already forbidden. In the United States, the two States of California and Michigan passed state laws to ban battery cages. Although some farmers who have to stop using battery cages will turn to enriched cages, many farmers will switch to loose housing systems. The resemblance of organic systems to other loose housing systems and the expected growth of the alternative egg market, make our results valuable for a larger group of stakeholders than the organic sector alone.

The most recent overview of how Dutch organic farms perform in terms of health and welfare is based on data from 2001 and 2002 [4]. The results of that study were obtained through manure samples and a questionnaire for farmers. No physical assessment of animals took place. The mean flock size was 1,840 (80–5,400), the mean mortality was 11.4% (0–21) and the main health problems were predators, piling, coli, endoparasites, infectious bronchitis, and brachyspira. In the United Kingdom, a 7% mean mortality was reported in 1997 as well as cannibalism and coccidiosis [5]. A German study in 1999 [6] that involved a physical assessment of slaughterhouse hens reported pododermatitis, keel bone deformations, endoparasites, and fatty livers. In 2001 a Swedish study [7] based upon a questionnaire for farmers with 12–1,700 hens found a 9% (1–60) mean mortality, cannibalism, endoparasites, red mites, and leg parasites as health problems. A 2001 study in Austria [8] found a 7.2% (0–32) mean mortality, endoparasites, infectious bronchitis, Salmonella and cannibalism as health problems in 500–700 bird flocks. A Danish study [9] of 18 flocks with 1,200–5,000 hens found a 22% (9–62) mean mortality that was mainly caused by Pasteurella, predatory attacks, and piling. Thirty-three percent of the Danish flocks had little or no feather damage and 22% had severe feather damage. Of course, the type of health problems reported depend on the research methods applied. Interviews with farmers reveal mortality, and physical assessment may reveal pododermatitis and keel bone deformities, whereas manure samples give information about endoparasites. Though this makes the studies mentioned difficult to compare, they do give an impression of the range of health and welfare issues for organic laying hens during a period when flock sizes were much smaller and there was no common European organic poultry keeping legislation. 

Organic hen keeping has been subjected to many changes, both in terms of regulations and trends in poultry husbandry since the most recent publication in 2003 [4] about the health and welfare of Dutch organic laying hens. Since then, beak treatments have been banned, aviaries have been introduced next to ground stables, flock sizes and number of hens per farm have increased, percentages of organic ingredients in poultry feed have increased, a national regulation for organic hen rearing has entered into force, different hybrids and genotypes have been introduced, and farmers have started to pay more attention to an attractive and functional free-range area. 

Furthermore, farmers with organic hens experienced higher mortality rates than those with barn systems and were keen to understand how the health and welfare of their animals could be optimized. They wanted to know which factors in the rearing period and laying period influenced animal health and welfare parameters. Research questions were: how are organic egg farms performing in terms of health and welfare? How are farm practices during rearing and laying related to hen health and welfare during the laying period? 

## 2. Methodology

We sent an invitation to 128 farms that kept organic laying hens according to Skal standards. Skal is the Dutch organic certification body. All farms that expressed an interest were included in the study. Our aim was for 50 flocks to take part. This number was a compromise between costs and statistic demands. In order to achieve a minimum of 50 flocks, farms who did not respond to the initial invitation were actively approached by phone in alphabetical order using the Skal’s address list. Once 50 flocks had joined the study, new flocks were no longer able to take part. The 25 rearing farms that were approached reared the ‘study flocks’. 

Different questionnaires were designed for egg farmers and rearing farmers. The questionnaires were based on questionnaires from earlier studies and were discussed with several experts: veterinarians, farmers, poultry researchers, the Dutch Animal Protection organization, poultry advisors, hatcheries, and the Ministry of Economic Affairs, Agriculture and Innovation. The questionnaires covered housing and management, hybrid type, flock size, number of birds per m^2^, feed, feed intake, egg production, mortality, mislaid egg percentage, feather pecking damage, health problems, and use of the free-range area. Both open and multiple-choice questions were included. The egg farmer questionnaires were filled in by the researcher during farm visits which took place when the hens were between 50 and 60 weeks old. Rearing farmers received their questionnaires by letter post and filled them out themselves.

A bird assessment protocol for farm visits was developed based on the following criteria:

- scoring should be objective,
- scoring should not demand expensive laboratory work,
- scoring items should be characteristics that farmers can see or feel themselves. This ensured that the study and its results were easy to communicate with the study’s target group, the farmers.

**Table 1:** Methods used for body condition scoring.

Indicator	Assessment method	Relevance to welfare
**Comb color**	Konica Minolta color reader CR-10; measures color and describes it using three parameters: L, A and B-value ^1^. L-value ranges from 0 to 100. A-values range from -86 to + 98. B-value ranges from -108 to + 94 ^2^.	Several diseases and health problems can cause a paler comb. Farmers regard bright red combs as a sign of health.
**Feather condition**	1. Visual scoring identifying a featherless spot of at least 5 centimeters diameter on a hen’s back (yes/no) 2. Visual scoring of flock pictures taken according to Tauson *et al.* [10]. Classification of scoring of 6 body parts on a scale from 1 (completely featherless) to 4 (no or few feathers missing) ^3^.	Featherless spots are caused by feather pecking. This is a sign of reduced welfare in both actor [11,12] and victim [13]. Feather pecking can be caused by several factors during the rearing and laying period.
**Wounds**	Visual scoring of wounds on back, vent and tail area (yes/no)	Skin wounds can be caused by pecking behavior or accidents. Wounds are a sign of reduced welfare and make a bird potentially more vulnerable to infections.
**Abnormalities on foot soles**	Visual scoring of foot soles by observer (normal/scab/abscess).	Foot soles can have wounds or infections, caused by abnormalities in housing and reduced resistance against diseases (see discussion).
**Keel bone deformations**	Palpation of keel bone: deformity yes/no. Deformities are defined as deviations from the normally straight line (lateral or dorso-ventral) or thickened sections.	Keel bone deformations can be caused by ‘metabolic bone disease’, accidents and resting on the keel during perches.
**Body weight**	Weighing scale in grams.	Body weight could be compared to breeding standards and uniformity within a flock could be calculated (see discussion).

^1^ Available online: http://sensing.konicaminolta.asia/learning-center/meter-measurement/meter-spaces (accessed on 2 April 2014); ^2^ Available online: http://stackoverflow.com/questions/19099063/what-are-the-ranges-of-coordinates-in-cielab-meter-space (accessed on 2 April 2014); 3 For each flock one mean was calculated for all body parts from 50 scored hens. To make the Tauson scores more illustrative, three ‘qualitative’ categories were defined: no/little damage (Tauson score 3.1–4); moderate damage (Tauson score 2.1–3) and severe damage (Tauson score 1–2).

The birds were assessed between 50 and 60 weeks of age. Fifty birds were caught individually at each farm. In tame flocks, this was done by hand while walking through the flock and in shy flocks by cornering the birds using a ‘catch crate’. The indicators listed in Table 1 were determined for each individual bird. To avoid inter-observer bias, bird assessments on all the farms were done by the same researcher.

A manure sample was obtained by collecting 20 fresh droppings indoors. These were mixed and sent to the GD Animal Health Service (Deventer, the Netherlands). The sample was analyzed using the McMaster flotation method for worm egg counts. Shell and yolk color measurements were done with a Konica Minolta color reader CR-10 on thirty first-grade eggs that were collected from each flock. Pictures were taken of flocks and housing. After individual birds were assessed, the fear level of the whole flock was assessed during the researcher’s visit. This was done on a scale from one (calm) to 10 (showing fearful behavior by flying up several times). The one, five and 10 on this scale were described and the observer made a rough estimate of the rest of the scale. Farmers scored health problem occurrence by indicating whether or not the flock had encountered a prelisted health problem (YES) in the flock or not (NO).

To estimate free-range use, the parameter Free Range Use (FRU) was calculated:

FRU = number of weeks free range is available (popholes open) in the period of 17–50 weeks of age × the maximum percentage of hens seen outside simultaneously under most optimal conditions (estimated by the farmer)

Since the flocks were 50–60 weeks old when we enquired about maximum range use, the percentages covered three seasons and all hours of the day. This prevented a ‘snapshot’ impression of range use.

All data were entered into an Excel spreadsheet. Statistics were calculated using the General Linear Model procedure (regression analysis). The RSEARCH procedure to find meaningful models (‘all subset regression’) was our starting point. Because R² was relatively low in most of these models, we moved on to linear regression for single factor relationships. In the regression analysis, relations were considered as not meaningful if the adjusted R² was less than 20. The two-sample *t*-test (unpaired, two-sided) and correlations (the two-sided test of difference from zero) were also used. GenStat for Windows, 13th edition, VSN International Ltd. (2010) was used to calculate all statistics.

## 3. Results

Results were collected from 49 organic laying flocks on 43 farms. Some of these farms volunteered after receiving our invitation letter. The rest joined when the letter was followed by a phone call. We received information about the rearing period of 35 of these 49 flocks.

### 3.1. Number and Size of Farms

Five of the 49 flocks were kept on four farms with less than 500 hens. Forty-four flocks were kept on 39 farms with more than 500 hens. The average number of hens on all farms was 9,300 (min 34–max 25,450). Table 2 shows the distribution of the number of hens per farm. 

Three egg farms had their own hen-rearing facilities.

**Table 2:** Distribution of farm sizes.

Size of farms	Number of farms	Percentage of farms
**0–100**	1	2
**101–500**	4	8
**501–1,000**	0	0
**1,001–5,000**	7	14
**5,001–10,000**	18	37
**10,001–15,000**	14	29
**15,001–20,000**	2	4
**20,001–25,450**	3	6
**Total**	49	100

### 3.2. Hybrids

The most-used hybrid was H&N Silver Nick (51%), followed by Hy-line Silver (20%), Hy-line Brown (10%) and Lohmann Brown Lite (8%). Most of the hens were so-called ‘silvers’: white- feathered hens that lay brown eggs. 

### 3.3. Housing and Use of the Free Range Area

Twenty-two (45%) of the 49 flocks were kept in aviaries and 27 (55%) in a floor-based housing system (see Table 3). The latter had a grid floor with perches above a manure pit. Eleven of the 22 aviaries had a winter garden, of which 10 were ‘regarded as stable area’. ‘Regarded as stable area’ means that the farmer includes its surface when calculating the number of hens allowed in the stable + winter garden combination. Such winter gardens do not provide additional space, but do provide ‘different’ space in terms of temperature and daylight. Twelve of the 27 floor-based stables had a winter garden, of which 11 were regarded as stable area. 

**Table 3:** Distribution of housing systems.

Housing system	No of flocks	Winter garden yes/no
**Aviary**	22	11 yes 11 no
**Floor**	27	12 yes 15 no
**Total**	49	23 yes 26 no

Thirty-nine (80%) of the 49 flocks had less than 25% sheltered area in the form of bushes, maize or artificial structures in their outdoor ranging area. Seven flocks (14%) had 26–50% sheltered area, and three flocks (6%) had more than 50% sheltered area (see Table 4). 19 out of 48 flocks (40 %) had mortality by predators: 7 flocks by birds of prey (15%), 6 flocks by foxes (13%) and 6 flocks by both (13%). 

**Table 4:** Amount of shelter in outdoor runs.

Percentage of surface covered with bushes, maize or artificial structures	Number of flocks	Percentage of flocks
**<25**	39	80
**26-50**	7	14
**>50**	3	6
**Total**	49	100

During the study, national avian influenza alerts resulted in five periods of decreed indoor confinement. Four of these periods were in autumn or winter and one of them was in the summer. Five of the 49 flocks were vaccinated against avian influenza. These flocks were allowed to range during the confinement periods. The hens that were allowed to range shortly after their arrival on the laying farm tended to range more between 50 and 60 weeks of age (correlation −0.33; *p* = 0.03). When hens were allowed to range, on average 62% of the birds in a flock were seen outside together. 

Flocks that used the outdoor run more intensively, with higher FRU, had better feather scores (regression analysis; R² = 25; *p* < 0.001). 

The more intensively hens used the outdoor run, the better farmers assessed their general health and performance (regression analysis; R² = 18; *p* = 0.003). 

### 3.4. Technical Performance

Table 5 is an overview of production parameters at 30 and 60 weeks of age.

**Table 5:** Production parameters at 30 and 60 weeks of age.

Item	30 weeks Mean (min-max)	60 weeks Mean (min-max)
**Feed intake (gram/hen/day)**	129 (109–147)	133 (113–160)
**Production (laying %)**	91 (76–96)	80 (58–92)
**Mislaid eggs (%)**	3 (0–12)	2 (0–12)
**Mortality (%)**	2 (0–11)	7.8 (0–34)

Zero % mislaid eggs means ‘close to zero’. Zero % mortality was found in one very small flock, where none of the hens died in their first 60 weeks. The one flock with 34% mortality was a 8,800-hen flock that got infected with the bacteria *Erysipelotrix*. 

### 3.5. Body Weight

Calculations were done for the H&N Silver Nicks because this was the only genotype with enough available flocks. We found a relation between growth during the period of 7 to 11 weeks and body weight in the laying period at 50–60 weeks (regression analysis; R² = 36; *p* = 0.005): the faster the pullets grew during the middle of the rearing period, the higher their body weight was at a later age. 

Table 6 shows mortality distribution.

**Table 6:** Distribution of mortality at 60 weeks of age.

Mortality	Number of flocks	Percentage
**0–5**	21	43
**6–10**	15	31
**11–15**	4	8
**16–20**	4	8
**>20**	1	2
**Unknown**	4	8
**Total**	49	100

Table 7 is a list of reported health problems. A health problem was defined as such if the farmer perceived it as a problem. Some problems were diagnosed by a veterinarian and some were not. Health problems were not quantified in terms of mortality or production loss. 

**Table 7:** Presence of health problems.

Health problem	Number of flocks
*E. Coli*	18 (37%)
Red blood mites	16 (33%)
Infectious Bronchitis	15 (31%)
Piling	15 (31%)
Skin infections	11 (22%)
Multi systemic wasting syndrome	11 (22%)
Intestinal parasites	9 (18%)
Chronic gut infection (enteritis)	6 (12%)
Blackhead	5 (10%)
Fatty livers	2 (4%)
Botulism	1 (2%)
Amyloidosis	1 (2%)
Coccidiosis	0 (0%)

### 3.6. Intestinal Parasites in Relation to Anthelmintic Use

For one flock it was unclear whether it had been wormed. Therefore, results were available for 48 flocks. Twelve of 48 flocks (25%) were not wormed up to 50 weeks of age (see Table 8). Thirty-one flocks (65%) were wormed irregularly or less frequently than recommended with an anthelmintic producer (e.g., Flubenol^©^ every 6 weeks). Only five flocks (10%) were wormed five or more times between 17 to 50 weeks of age. Table 8 shows egg count results. With regression analysis we did not find a meaningful relation between worming of a flock (yes/no) and % lay at 60 weeks (regression analysis; R² = 15.2; *p* = 0.005), or between worming of a flock (yes/no) and mortality at 60 weeks (regression analysis; R² = 6.2; *p* = 0.731). 

**Table 8:** Number of anthelmintic treatments up until 50 weeks of age (n = 48).

	Number of flocks	Positive for Ascaridia and Heterakis	Positive for Capillaria	Positive for Coccidiosis	Positive for Syngamus
**No treatment**	12 (25%)	83%	25%	25%	0%
**Irregular, less frequent than prescribed, *i.e.*, treated 1-4 times**	31 (65%)	61%	19%	42%	0%
**Treated 5 or more times**	5 (10%)	0 (0%)	0%	3 (60%)	0%

### 3.7. Parasites and Egg Yolk Color

Yolk color measurements determined L-, A- and B-values. In a color series of slightly yellow to orange, the L-value decreases and both the A- and B-values increase. Flocks that were positive for Ascaridia or Heterakis had higher L-values, meaning a lighter yolk color (*p* = 0.002; two-sample *t*-test; 47 d.f.). Flocks that were positive for Capillaria also showed a higher L-value, which meant a lighter yolk color (*p* = 0.035; two-sample *t*-test; 47 d.f.). 

### 3.8. Feather Pecking Damage

On average, 64% (minimum zero; maximum 100) of the hens in all 49 flocks had a featherless spot of at least five centimeters diameter on their backs. The score results using Tauson *et al.*’s [10] method of scoring six body parts of 50 hens are shown in Table 9. The scores between one and four were obtained following Tauson’s protocol. In addition we defined qualitative categories to make the outcomes more illustrative. Tauson’s scoring was done on the basis of photographs. Photographs that were of sufficient quality to score were available for only 37 flocks. 

**Table 9:** Severity of feather damage according to Tauson *et al.* (2005).

Category	Number of flocks
No/little feather damage (Tauson score 3.1–4)	25 (68%)
Moderate feather damage (Tauson score 2.1–3)	9 (24%)
Severe feather damage (Tauson score 1–2)	3 (8%)
**Total**	37

Twenty-five of 37 flocks (68%) had little or no feather damage, nine flocks (24%) had moderate damage and three flocks (8%) had severe damage. The feather score was better (RSEARCH procedure (all possible subset selection; R² = 52) if the FRU was higher (*p* = 0.003), the laying percentage at 60 weeks was higher (*p* = 0.007), and the sooner the hens went outside after arrival on the laying farm (*p* = 0.024). 

### 3.9. Vent Pecking

Peck wounds in the vent area were observed in 34 of 49 flocks (69%). On average, 18% (min two–max 50) of the birds in these 34 flocks had wounds in the vent area. It was not possible to make quantitative statements about wound severity as scores were based on wound presence (yes/no). Most of the wounds had an approximate diameter of 0.5 cm, a small amount was bigger (up to 2 cm diameter). 

### 3.10. Keel Bone Deformations

Keel bone deformations were found in all 49 flocks. On average, 21% (min four–max 48) of the birds in a flock had a keel bone deformation: a lateral or dorso-ventral deviation, a thickened section or a combination. In our study farmers used either round metal or rectangular wooden perches. With regression analysis we did not find a relation between keel bone deformation and perch type (regression analysis; R² = 11; *p* =0.012) or between keel bone deformation and aviary or floor-based housing (regression analysis; R² =11; *p* = 0.012). 

### 3.11. Foot Sole Lesions

Wounds on foot soles were observed in 38 of the 49 flocks (78%). On average, 13% (min two–max 48) of the birds in these flocks had such wounds. Since we scored hens on the basis of wound presence (yes/no), it is not possible to make quantitative statements on the severity of these wounds. The majority showed wounds of 0.2–0.5 cm diameter. Swellings that are characteristic for bumble foot were rarely observed. No relation was found between foot sole lesions and FRU (regression analysis; R² = 11; *p* = 0.013).

### 3.12. Comb Color

Comb color measurements entailed defining L-, A- and B-values. In a color series of pink to dark red, the L-value decreases and both A- and B-values increase. The hybrid type (with regression analysis) significantly explained the overall difference in comb color (L-value R² = 29, *p* = 0.003; A-value R² = 24; *p* = 0.009; B-value R² = 46, *p* < 0.001). Therefore, further calculations were only done for the most-used hybrid: H&N Silver Nick. A higher FRU was related to a lower L-value, thus darker combs (regression analysis; R² = 29; *p* = 0.003). Flocks that reacted more fearfully during our visits had higher L-values, thus lighter combs (regression analysis; R² = 34; *p* = 0.003). Flocks that tested positively for one or more intestinal parasites, tended to have a higher B-values, thus darker combs (*t*-test; *p* = 0.036). 

### 3.13. Rearing

Data regarding the rearing period was available for 35 of the 49 flocks. Not all questionnaires on the rearing period were complete. Six of the 33 flocks (18%) were kept in cages during the first weeks of their lives. Three of those flocks were kept on ‘pullet paper’ without litter. Twenty-six (79%) of the 33 flocks had litter from day one. In loose housed rearing, the group size was 13,800 (n = 25 flocks). The average pullet density during the first four weeks was 21 pullets/m^2^ (n = 33), with 8.5 as minimum and 33.5 as maximum. After four weeks, many flocks were given additional space, resulting in an average density of 14 pullets/m^2^ (min 6.6 and max 27.5). For 33 flocks, there was data available regarding whether they had access to an outdoor run. Nine flocks had not been outside and 26 flocks had access to an outdoor run. The average age for first time access was 8.4 weeks. At 17 weeks, on average 28% of the animals from the ‘outside flocks’ was seen outside. 

## 4. Discussion

The study incorporated data from 49 flocks on 43 farms. Some of the farms volunteered after receiving an invitation letter and the rest joined when the letter was followed by a phone call. It is possible that the farmers who volunteer to participate, are those who perform well. If this is the case, our results may overestimate the welfare situation on farms. Five of these 49 flocks were kept on four farms with less than 500 hens. Forty-four flocks were kept on 39 farms with more than 500 hens. According to Loefs [14] there were 107 farms with organic laying flocks in the Netherlands in 2008 and she only counted farms with more than 500 hens. Our 39 flocks represent slightly more than one third of the farms (39/107 = 36%).

### 4.1. Relation between Outdoor Run Use and Health and Welfare

We did not find any relation between the number of weeks that hens spent outside during the laying period and mortality at 60 weeks, nor was a relation found between the number of confinement periods and mortality at 60 weeks. However, several studies showed a higher mortality or a higher disease incidence in free-range or organic poultry compared to hens kept inside [15,16]. In 19 of 49 (39%) of our flocks there was mortality caused by predators. Other studies also reported mortality caused by predators. These studies focused on broilers [17] or they presented their results differently [18,19]. Therefore, it is not possible to compare these results to our results. However, all studies illustrate that losses caused by predators are a realistic risk of keeping free-ranging poultry. If mortality is a measure for bird welfare, then one could argue that hens on free-range farms have a reduced welfare. Flocks that used the outdoor run more intensively had better feather scores. If the amount of feather-pecking damage is used as a measure for hen welfare, then hens on farms where more hens use the free-range areas have a better welfare [20,21,22,23]. 

The more intensely hens used the outdoor run, the better the farmer assessed their general health and performance. Since we found this relation with regression analysis, there may not be a causal relation between the two parameters. Which health and performance aspects satisfy farmers regarding their hens remain unknown, but the relation shows that somehow good use of the outdoor run relates to good health and/or performance perceived by farmers.

### 4.2. Technical Performance

On average birds had a feed intake of 129 g/day (min 109–max 147) at 30 weeks and 133 g/day (min 113–max 160) at 60 weeks of age. The mean intake levels were within an acceptable range. The feed intake depends on ambient temperature, plumage condition, and housing system [24].

The mean number of mislaid eggs decreased during the laying cycle from three to two percent, which might be caused by adequate management interventions. The mean mortality of 7.8% at 60 weeks of age is difficult to compare with other studies, because these studies describe results of the entire laying cycle, which can be up to 70 weeks or more. Mortality is generally higher in alternative systems [25].

### 4.3. Body Weight

We found a relation between growth in the rearing period and body weight at 50–60 weeks: the faster the pullets grew during the middle of the rearing period (weeks of life 7–11), the higher was their body weight at later age. Although for several other aspects, a relation has been found between conditions in the rearing period and the laying period, for example feather pecking, we did not find literature specifically on laying hen body weight. 

### 4.4. Intestinal Parasites in Relation to the Use of Anthelmintic

Depending on the number of anthelmintic treatments, a considerable number of flocks tested positively for internal parasites. We did not find a relation with production or mortality. For different reasons it is not clear whether zero tolerance of internal parasites is necessary. Firstly, a low infection level might not be harmful. Secondly, when a higher infection level is combined with other health problems, one could ask what came first: the other health problems, which made the birds vulnerable to parasites or the parasites that made the birds vulnerable to other health problems. In 1999, Thamsborg *et al.* [26] already found that the significance of infections in terms of disease and production losses on organic farms had not been assessed. Moreover, the GD Animal Health Service, a Dutch authority on farm animal health, has the following text on their website [27] ‘not all internal parasite species are equally harmful’ and ‘treatment therefore is not always necessary’. They refer to ‘your personal vet’ for personal advice. Flubendazole is the only registered anthelmintic medicine against internal parasites in poultry, and parasites may become resistant to it. Therefore, zero tolerance in healthy flocks does not seem logical.

Flocks that were positive for Ascaridia or Heterakis had a lighter yolk color. Flocks that were positive for Capillaria also had a lighter yolk color. Although non-scientific on-line publications recognize this relation, we were unable to find scientific articles to support our findings. A general explanation seems to be that parasites damage the intestines, which in turn influences pigment intake.

### 4.5. Feather Pecking Damage

Sixty-four percent of the hens in all 49 flocks were found to have feather damage on their back when the first method was used. However, we found that 68% of the flocks had ‘no or little’ feather damage when Tauson’s method was applied in combination with categories we defined to qualify the damage. The difference may be caused by the fact that a five-centimeter featherless spot on a hen’s back qualifies as little damage if the rest of the hen’s feathers are in good condition, which was mostly the case. As Tauson’s method reflects variation better, we think that the method is more suitable than a quick scan of only one body part. 

Twenty-five of 37 flocks (68%) showed no or little feather damage, nine flocks (24%) showed moderate damage and three flocks (8%) showed severe damage. The feather score was better if the free-range use was better, the laying percentage at 60 weeks was higher, and the sooner the hens went outside after arrival on the laying farm. A relation between better free-range use and better feather cover is also reported in other studies [20,21,22,23]. A free-range area can be considered as environmental enrichment. Other types of environmental enrichment, such as additional foraging materials [28], scattered grains [29], and elevated perches [30] are also associated with less feather pecking. Another explanation could be that if a flock is distributed over a larger area, the stocking density decreases. Lower stocking density (in combination with a smaller group size) is also associated with less feather pecking [31,32,33]. Concerning the relation between higher laying percentage and better feather cover, it is not clear if this is a causal relationship. There is not much literature to be found on this relation, although Hüber-Eicher and Sebö [34] found the same relation in Swiss commercial flocks.

### 4.6. Vent Pecking

In 34 of 49 flocks (69%) peck wounds were seen in the vent area. On average, 18% (min two–max 50) of the birds had wounds in the vent area. Most of them were about 0.5 cm in diameter, a small number of them was bigger, up to 2 cm. Pötzsch *et al.* [35] found that 36.9% of non-cage housed flocks in the UK were affected by vent pecking. However, this was based on the farmer’s judgments obtained through voluntary questionnaires. Vent wounds are best observed by manually diagnosing individual hens. Lambton *et al.* [36], who did behavioral observations in 119 British flocks (barn, free-range and organic), observed vent pecking behavior in 24.8% of the flocks. According to Pötzsch *et al.* [35], vent pecking and feather pecking damage may be caused by shared common environmental risk factors.

### 4.7. Keel Bone Deformations

Keel bone deformations were seen in all 49 flocks. On average, 21% (min four–max 48) of the birds in a flock had a keel bone deformation: a lateral or dorso-ventral deviation, a thickened section or a combination. We are not sure how many of the deviations we observed were fractures, since we used the palpation method for our assessment instead of animal dissection. Wilkins *et al.* [37] compared palpation and dissection methods and concluded that in 91% of the cases palpation gave a correct result, assuming that the dissection method is the correct result. We are not sure whether the lateral and dorso-ventral deviations were the result of fractures or a slowly-developed curve. Wilkins *et al.* [37] found keel bone fractures in 36% to more than 80% of the animals in flocks kept in different housing systems. Gregory and Wilkins [38] found that 30% of 72-week old hens had a broken keel. Nicol *et al*. [39] found 52–59% of hens with keel breaks in commercial flocks in aviaries. In those studies, keel bone assessment was done on dissected hens. Compared to those studies, our 21% average of hens with a keelbone deviation (not all of them being a fracture) seems low. Twenty-one percent might be an underestimation of the real number of fractures. 

In our study, farmers used either round metal or rectangular wooden perches. We did not find any relation between keel bone deformation and perch type. Nor was a relation found between keel bone deformation and aviary or floor-based housing systems.

### 4.8. Foot Sole Lesions

In 38 of 49 flocks (78%) wounds on foot soles were seen. On average, 13% (min two–max 48) of the birds in these flocks had foot sole wounds. The majority of them were 0.2–0.5 cm in diameter. Swellings characteristic of bumble foot were rarely observed. 

Nicol *et al.* [39] measured foot damage in laying hens. They used a five-point scoring system with one meaning ‘no lesions’ and five meaning ‘very poor foot condition with inflamed and/or bleeding lesions visible over much of the area’. The foot damage in their study never exceeded an average value of 1.5, thus staying between ‘no lesion’ and ‘lesions which are clearly visible but of minor importance and/or frequency’. Although different assessment methods were applied, which means their results are difficult to compare with our results, we assume the results are about the same. 

Tauson and Abrahamsson [40] showed that perch design was an important factor for bumble foot in laying hens. Hens in cages without perches had the best scores: no bumble foot at all. When hens from cages with four different perch designs were compared, those with the narrow perches had the best bumble foot scores and those with broader perches the worst. The narrow perches were narrow rectangular (35 mm wide) or flat/round (38 mm wide). The broader perches were plastic ‘mushroom’ (48 mm wide) and wooden wide rectangular (53 mm wide). The authors’ explanation is that a good anatomical grip is not possible on broader perches and this results in too much pressure on the hen’s foot at rest. Lay *et al.* [25] concluded that wet litter conditions and high ammonia litter content in litter could cause footpad dermatitis in hens, which could be followed by bacterial infections, which in turn could lead to bumble foot due to penetration of ‘a strange body’. However, we also saw foot wounds in hens on dry litter. Perhaps the wet conditions of the free-range area added to the foot problems or it was caused by perch design in these hens as was found by Tauson and Abrahamsson [40]. According to their results, both the rectangular and the round perches in our study did not meet the hens’ needs.

### 4.9. Comb Color

The hybrid significantly explained the overall difference in comb color. Therefore, further calculations were only done for the most-used hybrid: H&N Silver Nick. FRU was related to darker combs. Flocks that were more fearful during our visits had lighter combs, but we could not find comparable results in the literature. Flocks that tested positively for one or more intestinal parasites tended to have darker combs. It is not clear whether this is a causal relation, because comb color in free-living red grouse is lighter in birds infected with internal parasites [41]. Spending more time in the free-range area is related to darker combs. A comparable finding is reported by Whay *et al.* [42].

## 5. Conclusions

With a mean flock size of 9,300 hens and 107 farms in 2008, the organic poultry sector has shown that it is able to become a serious alternative for existing intensive animal production. The presence of a free-range area is the most characteristic aspect of the organic system. Our study shows that a flock’s welfare benefits from using this free-range area, as is shows less feather pecking damage if it uses it. Concerning the presence of feather pecking damage and thus animal welfare, the organic system has the potential to perform better than ‘indoor’ poultry husbandry systems because of the presence of the free range area. The organic flocks perform about the same or worse than other commercial systems for several other factors. Improvements are desirable for mortality, internal parasites, keel bone deformations, and foot sole lesions. However, it can be questioned whether these physical consequences can be attributed to the housing system or if they are the result of the interaction between genotype and environment. The organic system uses the same high productive (and perhaps thus vulnerable) genotypes as other commercial systems in an environment that is still artificial.
